# Inaccurate offset restoration in total hip arthroplasty results in reduced range of motion

**DOI:** 10.1038/s41598-020-70059-1

**Published:** 2020-08-06

**Authors:** Markus Weber, Christian Merle, Danyal H. Nawabi, Sebastian Dendorfer, Joachim Grifka, Tobias Renkawitz

**Affiliations:** 1 Department of Orthopaedic Surgery, Asklepios Klinikum Bad Abbach, Regensburg University Medical Center, Kaiser-Karl V.-Allee 3, 93077 Bad Abbach, Germany; 2 Department of Orthopaedic and Trauma Surgery, Heidelberg University Medical Center, Heidelberg, Germany; 3 Orthopaedic Surgery and Sports Medicine, Hospital for Special Surgery, New York, USA; 4 Faculty of Mechanical Engineering, Laboratory for Biomechanics, Ostbayerische Technische Hochschule, Regensburg, Germany

**Keywords:** Bone imaging, Adaptive clinical trial

## Abstract

Offset restoration in total hip arthroplasty (THA) is associated with postoperative range of motion (ROM) and gait kinematics. We aimed to research into the impact of high offset (HO) and standard stems on postoperative ROM. 121 patients received cementless THA through a minimally-invasive anterolateral approach. A 360° hip ROM analysis software calculated impingement-free hip movement based on postoperative 3D-CTs compared to ROM values necessary for activities of daily living (ADL). The same model was then run a second time after changing the stem geometry between standard and HO configuration with the implants in the same position. HO stems showed higher ROM for all directions between 4.6 and 8.9° (p < 0.001) compared with standard stems but with high interindividual variability. In the subgroup with HO stems for intraoperative offset restoration, the increase in ROM was even higher for all ROM directions with values between 6.1 and 14.4° (p < 0.001) compared to offset underrestoration with standard stems. Avoiding offset underrestoration resulted in a higher amount of patients of over 20% for each ROM direction that fulfilled the criteria for ADL (p < 0.001). In contrast, in patients with standard stems for offset restoration ROM did increase but not clinically relevant by offset overcorrection for all directions between 3.1 and 6.1° (p < 0.001). Offset overcorrection by replacing standard with HO stems improved ROM for ADL in a low number of patients below 10% (p > 0.03). Patient-individual restoration of offset is crucial for free ROM in THA. Both over and underrestoration of offset should be avoided.

## Introduction

Although total hip arthroplasty (THA) is a highly successful treatment option for end-stage osteoarthritis with a positive responder rate of over 90%^1^^,^ the surgeon is faced with high patients’ expectations regarding the functional capacity of the artificial hip joint. Beside a correct intraoperative orientation of both cup and stem, restoration of biomechanics such as offset is crucial for optimal function and long-term outcome after THA^2–4^. Failure of correct offset restoration is associated with impingement, reduced hip abductor strength, altered gait kinematics and even higher wear of the artificial hip joint^5–8^.

To address the interindividual variability of the femoral anatomy, most modern implant systems offer at least two different offset geometries of the femoral stem—a standard offset design and a high offset design^9^. Prior to surgery the biomechanical restoration of offset is usually templated on radiographs illustrating the preferred stem design for the respective patient. However, intraoperative alterations in relation to the preoperative plan or reduced joint stability harbour the potential to complicate the right choice of femoral offset design during THA. The discussion of correlation between high offset (HO) stems and higher range of motion (ROM) is controversial in literature. Although aiming to find the balance between the risks of impingement due to offset reduction on the one hand and potential trochanteric pain syndrome in case of offset overrestoration on the other hand some studies prefer high offset designs intending to increase soft tissue tension and hip stability^10,11^ whereas others do not see a benefit in range of motion for high offset stems^12^. In detail, Shoji et al. report an increase in flexion of 15° and in internal rotation of 10° due to an increase in offset of 8 mm^10^. In contrast, Hayashi et al. found an increase of 2° regarding flexion and no difference regarding internal rotation when using a high stem geometry^12^. However, these studies do not account for the exact offset restoration in terms of offset over and underrestoration. To the best of the authors’ knowledge no study has analysed the impact of offset over- and underrestoration on postoperative range of motion ROM comparing a standard with a high offset stem geometry using postoperative 3D-CT so far.

The current study aimed to compare free ROM without osseous and/or prosthetic impingement between femoral stems with standard or high offset neck geometry in minimally invasive THA using a custom made 360° hip ROM analysis software. We hypothesized that the use of stems with a high offset geometry would result in higher ROM. In particular, offset over- and underrestoration was simulated and its effect on ROM regarding activities of daily living (ADL) analysed.

## Patients and methods

In the course of a registered, prospective controlled trial (DRKS00000739, German Clinical Trials Register) three dimensional computed tomography scans (3D-CT) were obtained after minimally-invasive cementless THA. The investigation was approved by the local medical ethics committee of the University of Regensburg (No.: 10-121-0263). All methods were performed in accordance with the relevant guidelines and regulations. A written informed consent was obtained from all participants of this study. The original study dealt with comparing navigated to free hand THA^13^. The current study is an independent analysis of the postoperative 3D-CT data.

**Figure 1:**
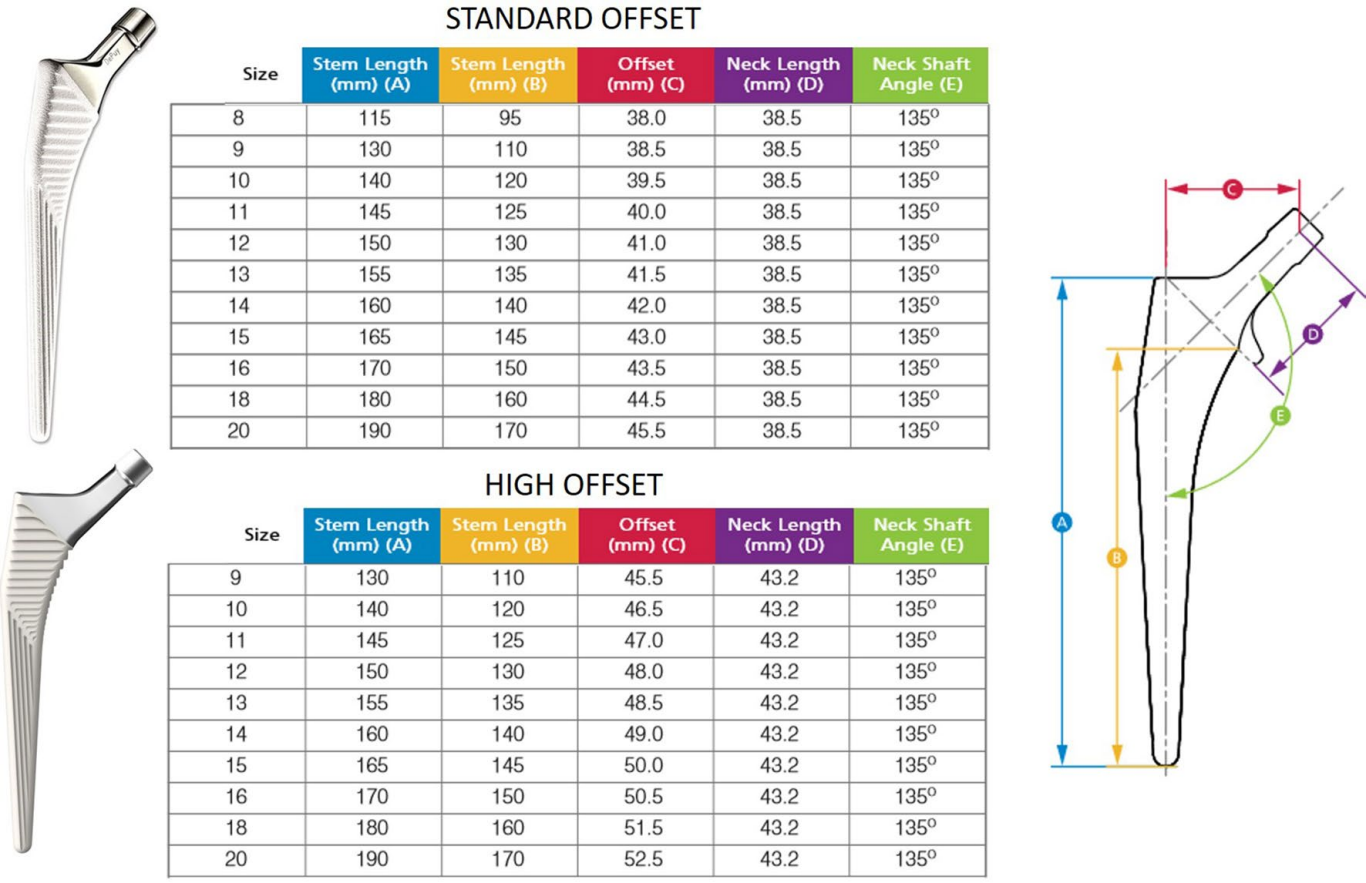
Implant design of the Corail stem with standard and high offset geometry (with kind permission of ©Johnson & Johnson Medical GmbH, DePuySynthes).

According to the study protocol, eligible participants were patients between the ages of 50 and 75 with an American Society of Anaesthesiologists (ASA) score ≤ 3 who were admitted for primary cementless unilateral THA due to primary or secondary osteoarthritis. THA in all patients was performed in the lateral decubitus position using a minimally-invasive single-incision anterolateral approach by four experienced orthopaedic senior surgeons^14^. In all patients press-fit acetabular components with neutral liners and cement-free hydroxyapatite-coated stems (Pinnacle cup, Corail stem, DePuy, Warsaw, IN, USA) with metal heads of 32 mm diameter were used (neck shaft angle 135°, cone 12/14, head neck ratio 3.50 for extension/flexion and 2.66 for abduction/adduction). The offset of the femoral stem increases with stem size and ranges from 38.0 to 45.5 mm for the standard offset geometry and from 45.5 to 52.5 mm for the high offset version. For all stem sizes the difference in femoral offset between the neck configuration with standard offset and HO is 7 mm (Fig. 1). The design is a straight, tapered cementless stem that fills the metaphysis and proximal diaphysis in the mediolateral plane. The final position of the “best-fitting” stem is a compromise of fitting a straight stem down the canal of the femur, addressing the flexion and twist of the proximal femur. Intraoperatively after osteotomy of the femoral neck and removal of the head, the femur was exposed. Then the medullary canal was reamed using broaches of ascending size, until the broach reached a stable position. Intraoperative fluoroscopy was used to control the size as well as the “best fitting” position of the broach regarding flexion and torsion of the femur according to two radiographic planes. The stem was inserted in the most stable position predefined by the patient-individual anatomy^15^. According to the patient’s individual anatomic situation offset was restored by using either a stem with a standard offset or a stem with a high offset geometry (HO). As a reference, the non-affected contralateral side was used and offset restored with the help of digital planning software (mediCAD,Hectec GmbH, Landshut, Germany). Six weeks postoperatively, a pelvic/femoral 3D-CT was performed (Somatom Sensation 16; Siemens, Erlangen, Germany).

In total, 121 data sets were included for final analysis (Table 1). Independent manual CT segmentation was performed on the pelvic bone and on the metal acetabular and femoral components by an independent external institute (Fraunhofer MEVIS, Bremen, Germany), blinded to individual patient data. Additionally, reference landmarks for providing the pelvic and femoral coordinate system were defined. This included both ASIS and pubic tubercle points to define the pelvic coordinate system and femoral head centre, mechanical axis and condyle axis to define the femoral coordinate system^16^. Based on the manually segmented bone models, a custom made 360° hip ROM analysis was performed by a previously described algorithm which automatically determines osseous and prosthetic impingement by virtually moving the leg until a collision between the 3D objects occurs^2,13,17^. The high accuracy of this 3D-CT method with deviations in ROM measurements of 1° was demonstrated in a previous study^16,18^. For every single patient ROM analysis was performed twice, first using the implanted stem geometry and second after replacing the original stem with the other offset configuration either standard or HO. For the alternative offset stem geometry the same size and position as obtained during the original implantation of the stem were chosen only changing the neck geometry between the standard and HO configuration. Since both stem geometries (standard and HO) share the same geometric configuration of the stem body filling the osseous femoral channel and differ only in terms of the prosthetic neck, the replacement can be easily obtained. In addition, the same head size and acetabular component were used for 3D-CT ROM calculations. Virtual 3D-ROM calculation were performed by moving the femur in all directions while the pelvis was set in a fixed position. The software recorded ROM boundaries until a collision between the femur and the pelvis occurred. In contrast to previous studies the software enabled to measure both collision between bone and or prosthesis due to the availability of the patient’s individual anatomic configuration in 3D-CT. Beside single ROM directions the proportion of patients was comparatively assessed reaching the hip joint ROM configurations without impingement for ADL as given by Davis et al., Miki et al. and Turley et al. with at least 110° of flexion, 30° of extension, 50° of abduction, 30° of adduction, 45° of external rotation during extension and 30° of internal rotation during 90° of hip flexion, respectively^19–21^ regarding combined prosthetic and osseous impingement.

**Table 1:** Anthropometric characteristics of the study group (n = 121).

Gender (female)	64 (52.9%)
Age (years)	62.7 (SD 7.6)
BMI (kg/m^2^)	27.1 (SD 4.2)
**Treatment side (right)**	65 (53.7%)
ASA 1	24 (19.8%)
ASA 2	63 (52.1%)
ASA 3	34 (28.1%)
Kellgren–Lawrence-Score	8 (5–10)

For categorical data values are given as relative and absolute frequencies, for quantitative data values are given as mean (standard deviation) or median (range).

*SD* standard deviation, *ASA* American Society of Anaesthesiology Score.

For statistical analysis, normally and nonnormally distributed continuous data are presented as mean (standard deviation) or median (range), respectively. Group comparisons were performed by Wilcoxon-tests on a 5% significance level for dependent variables. Absolute and relative frequencies were given for categorical data and compared between groups by McNemar tests on a 5% significance level. IBM SPSS Statistics 25 (SPSS Inc, Chicago, IL, USA) was used for analysis.

## Results

Intraoperative characteristics of stem geometry as well as implant sizes and position of the study group are demonstrated in Table 2. Stems with standard neck offset were chosen for optimal restoration of offset in 49.6% (60/121) of patients and stems with a HO geometry were implanted in 50.4% (61/121) of patients, respectively. Regarding the accuracy of biomechanical restoration, femoral offset restoration within a target zone of 5 mm succeeded in 90.9% (110/121), global offset in 91.7% (111/121) and leg length in 89.3% (108/121) of patients compared to the contralateral side, respectively.

**Table 2:** Intraoperative characteristics of the study group (n = 121).

Cup size	54 (48–62)
Femoral component size	12 (9–16)
Cup inclination (°)	42.4 (SD 5.8)
Cup anteversion (°)	17.9 (SD 8.1)
Femoral anteversion (°)	8.0 (SD 9.6)
Cup coverage (%)	87.6 ( SD 9.3)

Quantitative data values are given as mean (standard deviation) or median (range).

*SD* standard deviation.

**Figure 2:**
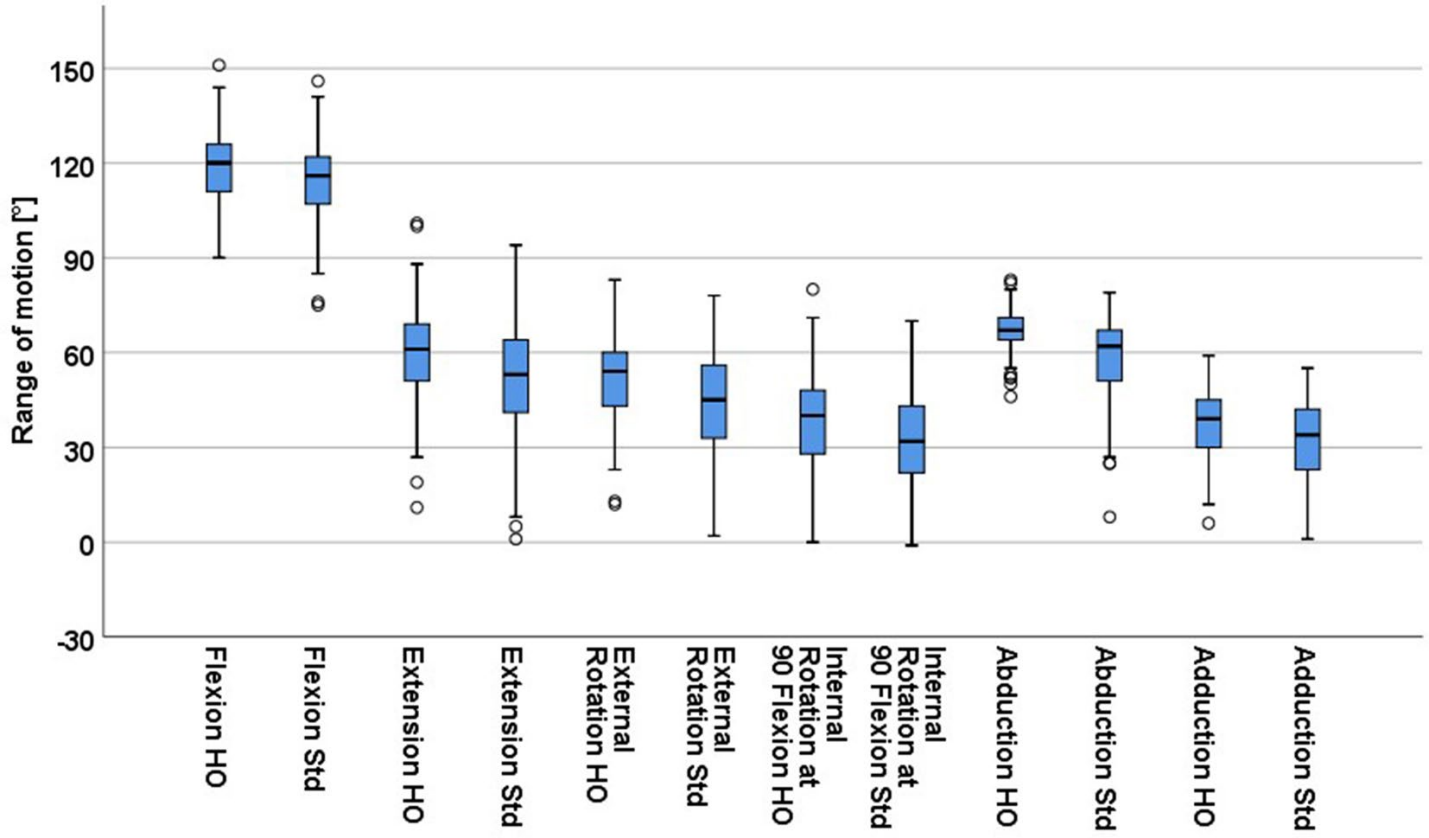
Comparison of mean range of motion between high offset (HO) and standard offset (Std) femoral stem.

**Figure 3:**
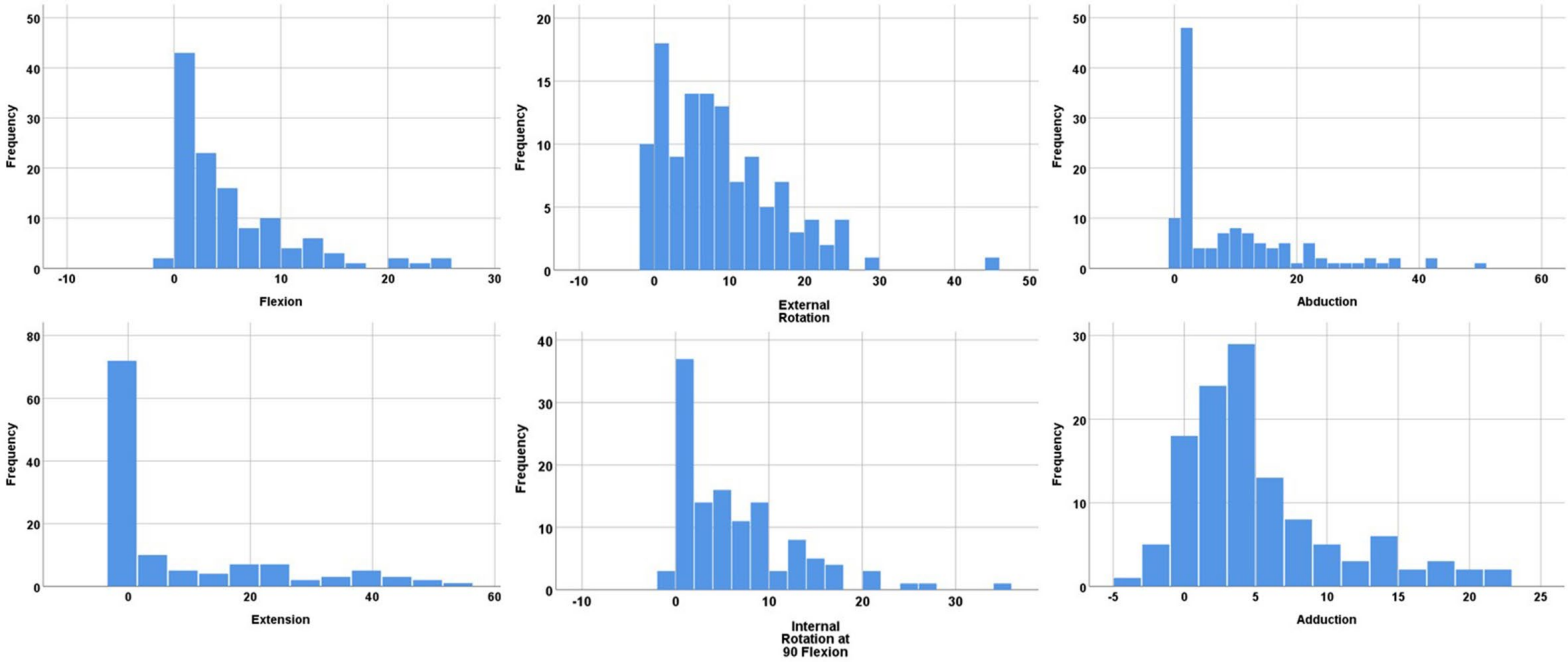
Distribution of differences in range of motion between high offset and standard stem.

Comparison of ROM analysis for each patient between the HO and standard offset stem configuration revealed higher values for the stem geometry with a HO neck regarding all ROM directions particularly of 4.6° (5.6°, p < 0.001) for flexion, 8.9° (14.2°, p < 0.001) for extension, 8.3° (7.9°, p < 0.001) for external rotation, 6.0° (6.4°, p < 0.001) for internal rotation at 90° flexion, 8.9° (10.7°, p < 0.001) for abduction and 4.7° (5.5°, p < 0.001) for adduction (Fig. 2). However the change in ROM between standard and HO stem was highly variable and differed widely from patient to patient ranging from − 1° to 15° for flexion, − 1° to 44° for extension, − 1° to 21° for external rotation, − 1° to 14° for internal rotation at 90° flexion, 0° to 28° for abduction and − 2° to 22 for adduction (Fig. 3).

**Figure 4:**
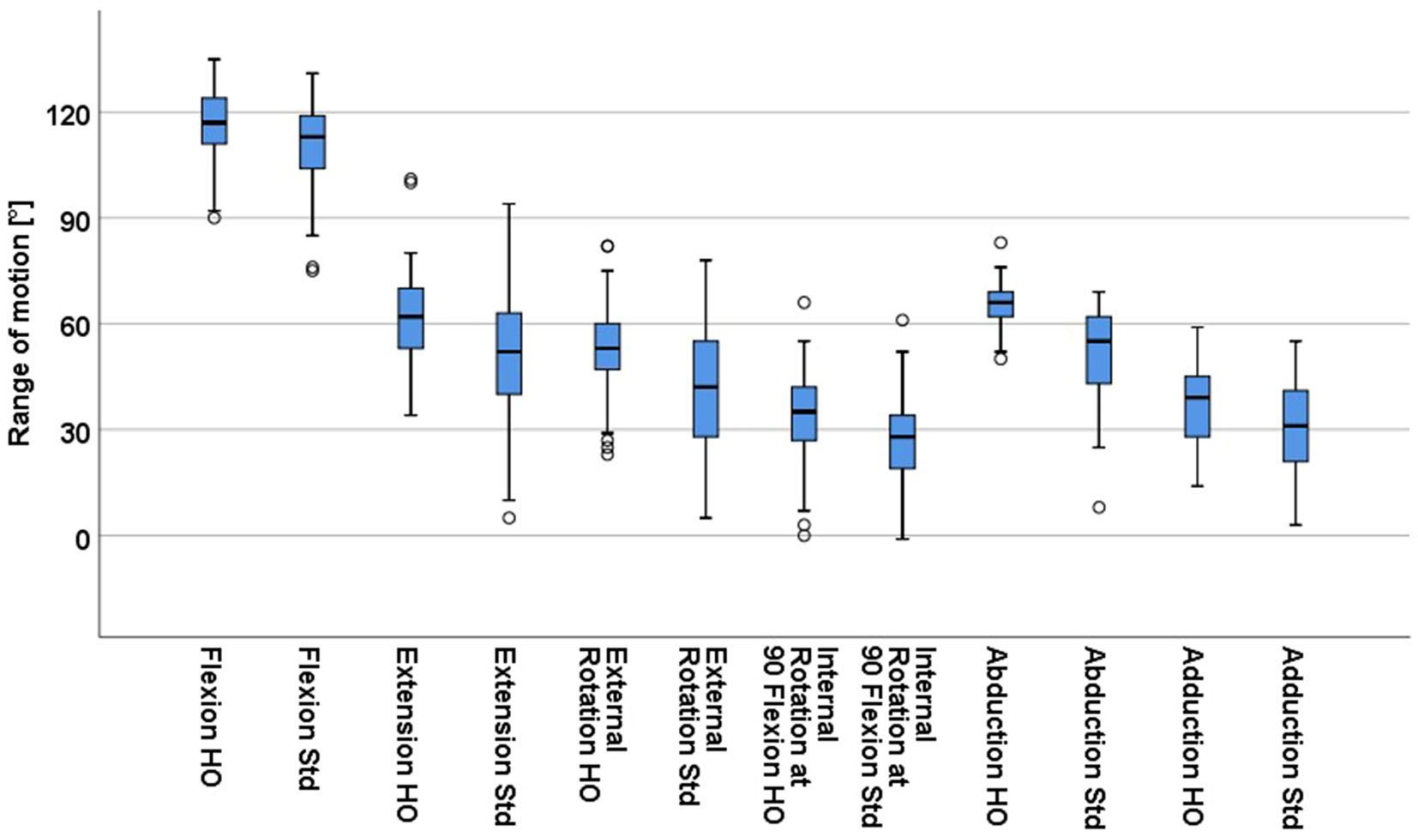
Impact of offset underrestoration by replacing the high offset (HO) stem with the standard offset (Std) stem in the subgroup with implanted high offset stems.

Based on these results the question came up which patients benefited from a HO stem. Therefore, we focused on the subgroup with implanted HO stem geometries for biomechanical offset restoration and calculated the change in range of motion if a standard stem had been used in these patients instead. Underrestoration of offset led to a decrease of 6.1° (6.7°, p < 0.001) for flexion, 11.5° (15.1°, p < 0.001) for extension, 12.1° (8.4°, p < 0.001) for external rotation, 8.1° (7.5°, p < 0.001) for internal rotation at 90° flexion, 14.4° (11.7°, p < 0.001) for abduction and 6.2° (5.9°, p < 0.001) for adduction (Fig. 4). Consequently a lower rate of patients fulfilled the ROM criteria required for ADL with reduction of 21.3% (13/61, p < 0.001) for flexion, 13.1% (8/61, p = 0.01) for extension, 36.1% (22/61, p < 0.001) for external rotation, 26.2% (16/61, p < 0.001) for internal rotation at 90° flexion, 41.0% (25/61, p < 0.001) for abduction and 23.0% (14/61, p < 0.001) for adduction (Table 3).

**Table 3:** Number of patients in percentage fulfilling ROM criteria for ADL in the HO group.

N = (61)	Flexion	Extension	External rotation	Internal rotation	Abduction	Adduction
HO	80.3 (49/61)	100 (61/61)	77.0 (47/61)	67.2 (41/61)	100 (61/61)	73.8 (45/61)
Std	59.0 (36/61)	86.9 (53/61)	41.0 (25/61)	41.0 (25/61)	59.0 (36/61)	50.8 (31/61)
p-value	< 0.001	0.01	< 0.001	< 0.001	< 0.001	< 0.001

*ROM* range of motion, *ADL* activities of daily living, *HO* high offset stem, *Std* standard offset stem.

**Figure 5:**
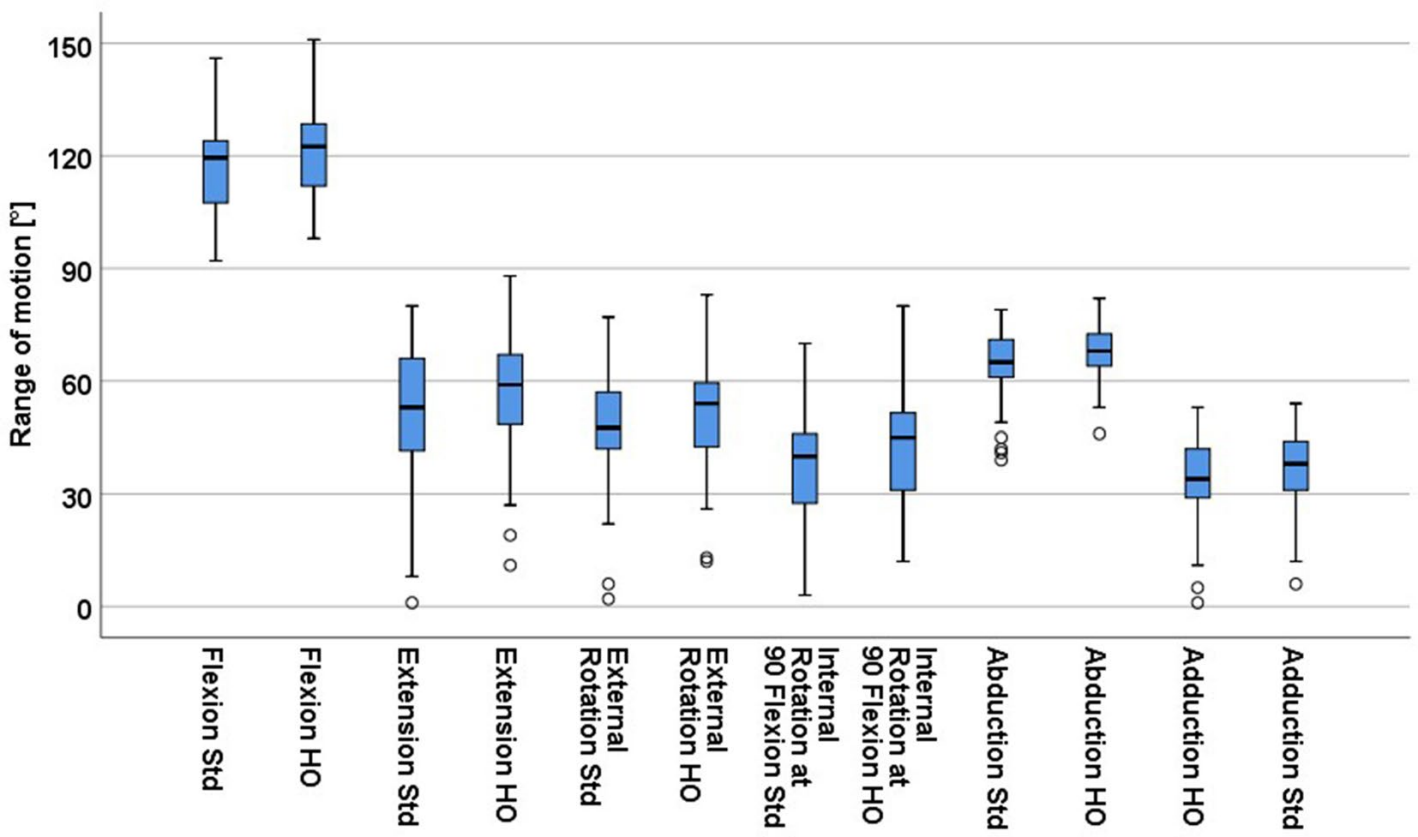
Impact of offset overrestoration by replacing the standard offset (Std) stem with the high offset (HO) stem in the subgroup with implanted standard offset stems.

Concentrating on the subgroup with implanted standard  stems for correct offset restoration we analysed change in ROM if the stem with the standard offset geometry had been replaced with a HO stem. Overrestoration of offset showed an increase of 3.1° (3.6°, p < 0.001) for flexion, 6.1° (12.7°, p < 0.001) for extension, 4.5° (5.0°, p < 0.001) for external rotation, 3.8° (4.2°, p < 0.001) for internal rotation at 90° flexion, 3.4° (5.3°, p < 0.001) for abduction and 3.2° (4.6°, p < 0.001) for adduction (Fig. 5). Correspondingly the rate of patients with free ROM of ADL increased by 6.7% (4/60, p = 0.13) for flexion, 10.0% (6/60, p = 0.03) for extension, 8.3% (5/60, p = 0.13) for external rotation, 5.0% (3/60, p = 0.25) for internal rotation at 90° flexion, 6.7% (4/60, p = 0.13) for abduction and 3.4% (2/60, p = 0.63) for adduction (Table 4).

**Table 4:** Number of patients in percentage fulfilling ROM criteria for ADL in the Std group.

N = (60)	Flexion	Extension	External rotation	Internal rotation	Abduction	Adduction
Std	71.7 (43/60)	85.0 (51/60)	61.7 (37/60)	71.7 (43/60)	91.7 (55/60)	73.3 (44/60)
HO	78.3 (47/60)	95.0 (57/60)	70.0 (42/60)	76.7 (46/60)	98.3 (59/60)	76.7 (46/60)
p-value	0.13	0.03	0.13	0.25	0.13	0.63

*ROM* range of motion, *ADL* activities of daily living, *HO* high offset stem, *Std* standard offset stem.

## Discussion

The purpose of this study was to determine the impact of offset over- and underrestoration on postoperative free ROM without osseous and prosthetic impingement by comparing standard offset stems with high offset stems in THA. In a 360° hip 3D-ROM analysis we found offset underrestoration resulted in a decrease of over 20% (p < 0.001) of patients fulfilling ROM criteria required for ADL, whereas overrestoration of offset lead to negligible increase of less than 10% (p > 0.03) in patients with free ROM for ADL.

There are several limitations of this study. First, the 3D-CT based ROM analysis enabled measurement of combined prosthetic and/or osseous impingement. However, it was not possible to account for soft tissue impingement. In more obese patients soft-tissue restrictions may limit ROM boundaries postoperatively. However, after THA all requirements regarding free ROM should be met independently of soft tissue restrictions, since the soft tissue situation might change over time. Second, the influence of pelvic tilt was not inlcuded in the current study. Although previous studies describe an impact of pelvic tilt on implant position and thus postoperative ROM^22^^,^ the authors feel intraoperative assessment of the variability of dynamic pelvic tilt and spinopelvic balance has not been resolved yet^23^. Third, the results of the present study rely on the stem design of a single manufacturer. This stem type anchors on the metaphysis and proximal diaphysis following the anatomic twist and bow of the proximal femoral canal. The fixation and consequently the postoperative stem position might be different in other stem designs especially for short and/or anatomic stems which could potentially impact ROM differences between HO and standard geometries. For that reason, the results of this study are restricted to the current stem design, which is, however, one of the most frequently used cementless femoral components in modern THA (> 2,000,000 implanted stems in 2015)^24^. Fourth, analysis were performed by a 360° hip ROM analysis software using 3D-CT. Due to the methodology in this study, it was not possible to obtain comparative clinical data^25^ on outcome enabling a comparison between the offset overrestoration and underrestoration. Therefore, the results do not allow correlation to important clinical parameters or outcomes, such as dislocation, wear, or revision.

A strength of the study is the fact that postoperative implant position of both cup and stem was controlled on 3D-CT thereby minimizing confounding factors. Furthermore, biomechanical reconstruction accuracy such as leg length and offset also possibly affecting postoperative ROM was checked^3^. Any difference with regard to individual hip joint ROM in the current analysis is due purely to the change in stem geometry varying between standard and HO stem design. Theoretically, femoral stems with a modular neck could allow control of femoral version and varus/valgus angulation of the neck thus varying ROM^26,27^. However, such modularity has been the source of serious concern due to the potential release of metal ions^28^.

In answer to the first question of the study, femoral stems with a HO neck geometry showed higher ROM values between 5° and 9° for all directions. Although no sovereign power calculation was performed, the statistical significance of the observed differences indicates appropriate power. Within the study cohort a high interindividual variability was observed. In some patients the use of a HO stem resulted in an increase in ROM of over 20°, whereas in other patients no improvement in ROM was measurable. Comparing the results to the literature, a previous saw bone study recommended high offset neck designs to improve ROM. Single increase in offset by choosing a different modular neck correlated with higher flexion up to 20° and higher extension up to 15° using a head diameter of 32 mm. Similarly, internal rotation was reported to increase with offset but the effect was smaller and depended on the neck shaft angle. However, external rotation decreased with additional neck offset of the femoral stem due to neck on liner impingement^11^. Correspondingly, a higher ROM was observed in a cadaveric study for the modular PerFix stem (Kyocera Medical, Osaka, Japan) with higher offset than the low offset version. An increase in offset of 8 mm resulted in 26.7° higher flexion and 21.2° higher internal rotation^29^. In contrast, another clinical study compared intraoperative ROM between standard offset and HO of the Summit stem (DePuy, Warsaw, IN, USA) using computed tomography based navigation. The authors found no correlation between stem offset and ROM except for higher external rotation^12^. The implant specific natural offset design with 36–44 mm was comparable to the stem used in this study. However, the neck shaft of this stem is 5° lower with 130° compared to the stem design of the current study which might at least partly explain the different results in relation to the present study. None of the previous studies accounted for offset restoration in relation to the standard or high offset stem geometry. Beside the differences in implant design this might further illuminate why the results of ROM analysis for HO stems are so controversial in literature.

Therefore, we built two subgroups and analysed the effect of offset overrestoration if the intraoperatively inserted standard offset stem was replaced by a high offset neck geometry and underrestoration if the intraoperatively chosen high offset stem was replaced by a standard offset stem, respectively. In the study cohort the distribution of standard and offset stems was equal. Sixty patients required a standard stem for offset restoration compared to the contralateral side and 61 patients received a HO stem geometry to equalize offset differences. In the 61 patients, underrestoration of offset by replacing the HO stem with the standard offset stem led to a substantial reduction of ROM for all directions with a mean change between 6° and 14°. The clinical relevance of this difference in ROM was demonstrated by a lower rate of patients that fulfilled ROM boundaries required for ADL. Offset underrestoration consequently led to decrease of over 20% of patients for each ROM direction that reached the required ROM for ADL except for extension with 13%. This means offset underrestoration reduces ROM within a critical range resulting in impingement and thus instability while performing typical motions in daily life. Therefore high offset stems represent an inevitable tool to restore offset in patients with corresponding anatomic configuration and thus help to ensure free ROM without impingement in a clinically critical range. On the other hand, excessive overcorrection as simulated in the 60 patients with originally implanted standard offset stems by using a HO stem increased ROM but not clinically relevant with mean values between 3° and 6°. This was demonstrated by a low percentage of patients that gained additional ROM required for ADL of fewer than 10% for all ROM directions. The slight but not relevant higher ROM by offset overrestoration is bought with the risk of increased strain in the medial cortex and thus potential early failure of the femoral component, higher polyethylene wear of the liner, altered gait kinematics and potential trochanteric pain syndrome^6,30,31^. This differentiated perspective of HO stems regarding offset over- and underrestoration might at least partly explain the controversial data situation according to the literature available where some studies report higher ROM for HO stem geometries^11,29^ while others see no benefit in ROM for HO stems^12^.

In conclusion high offset stems are an essential option to restore biomechanics and enable free ROM without impingement for ADL in a substantial proportion of patients undergoing THA. However, an overrestoration of offset with HO stems does necessarily appear to improve postoperative ROM in patients in whom standard offset stems allow for adequate offset reconstruction. The patient`s individual anatomy has to be accounted for during THA to restore the patient specific biochemical offset configuration. Both excessive offset under- and overrestoration should be avoided in THA.

## Data Availability

Detailed data information can be provided upon further request at University of Regensburg, Department of Orthopaedic Surgery, Bad Abbach Medical Centre.
